# Novel assessment of haemodynamic kinetics with acute exercise in a rat model of pulmonary arterial hypertension

**DOI:** 10.1113/EP085182

**Published:** 2015-06

**Authors:** Mary Beth Brown, Tsungai J. Chingombe, Abigail B. Zinn, Jagadeshwar G. Reddy, Rachel A. Novack, Sean A. Cooney, Amanda J. Fisher, Robert G. Presson, Tim Lahm, Irina Petrache

**Affiliations:** 1 Department of Physical Therapy, Indiana University School of Health and Rehabilitation Sciences, Indianapolis, IN, USA; 2 Pulmonary and Critical Care, Department of Medicine, Indiana University School of Medicine, Indianapolis, IN, USA; 3 Department of Anesthesiology, Indiana University School of Medicine, Indianapolis, IN, USA; 4 Richard L. Roudebush VA Medical Center, Indiana University School of Medicine, Indianapolis, IN, USA

## Abstract

Exercise improves outcomes of multiple chronic conditions, but controversial results, including increased pulmonary artery (PA) pressure, have prevented its routine implementation in pulmonary arterial hypertension (PAH), an incurable disease that drastically reduces exercise tolerance. Individualized, optimized exercise prescription for PAH requires a better understanding of disease-specific exercise responses. We investigated the acute impact of exercise on already-elevated PA pressure and right ventricular (RV) wall stress and inflammation in a rat model of PAH (PAH group, *n* = 12) induced once by monocrotaline (50 mg kg^−1^, i.p.; 2 weeks), compared with healthy control animals (*n* = 8). Single bouts of exercise consisted of a 45 min treadmill run at 75% of individually determined aerobic capacity (${\dot{V}}_{{\text{O}}_{\text{2}}\text{max}}$). Immediately after exercise, measurements of RV systolic pressure and systemic pressure were made via jugular and carotid cannulation, and were followed by tissue collection. Monocrotaline induced moderate PAH, evidenced by RV hypertrophy, decreased ${\dot{V}}_{{\text{O}}_{\text{2}}\text{max}}$, PA muscularization, and RV and skeletal muscle cytoplasmic glycolysis detected by increased expression of glucose transporter-1. Acute exercise normalized the monocrotaline-induced elevation in RV systolic pressure and augmented pulmonary endothelial nitric oxide synthase activation, without evidence of increased RV inflammation or apoptosis. Real-time recordings of pulmonary and systemic pressures during and after single bouts of exercise made using novel implantable telemetry in the same animal for up to 11 weeks after monocrotaline (40 mg kg^−1^) corroborated the finding of acute PA pressure decreases with exercise in PAH. The PA pressure-lowering effects of individualized exercise associated with RV-neutral effects and increases in vasorelaxor signalling encourage further development of optimized exercise regimens as adjunctive PAH therapy.

## Introduction

Pulmonary arterial hypertension (PAH) is a devastating disease of progressive remodelling of small- to medium-sized pulmonary arteries that leads to right ventricular (RV) failure and death (Archer et al. 2009). Clinically, the disease is characterized by poor functional status with marked decrease in physical capacity and hypoxaemia with activity or even rest (Archer et al. 2009). Despite the development of several pharmacological therapies for the disease, no cure exists. Until nearly a decade ago, exercise was discouraged for PAH. Since then, a growing body of literature indicates that aerobic exercise may be an important therapeutic strategy for PAH (Uchi et al. 2005; Mereles et al. 2006; de Man et al. 2009; Mainguy et al. 2010; Fox et al. 2011; Grünig et al. 2012; Zafrir, 2013), but the mechanisms for benefit have not been defined. In addition, concerns exist that exercise, if performed too vigorously, may lead to maladaptive changes in the RV (Handoko et al. 2009).

Data from healthy animals and models of systemic vascular disease suggest beneficial effects of exercise via upregulation of endogenous endothelial nitric oxide synthase (eNOS) expression and activity (Sessa et al. 1994; Shen et al. 1995; Johnson et al. 2001; Kojda & Hambrecht, 2005; Kuru et al. 2009; Zhou et al. 2010) and a subsequent increase in NO production (Miyauchi et al. 2003) and NO-dependent arterial relaxation (Johnson et al. 2001; Kuru et al. 2009; Gielen et al. 2011). As both NO production and NO-dependent arterial relaxation are typically impaired in patients with PAH (Konduri et al. 2003), eNOS-mediated adaptations after exercise training would be a plausible mechanism for the functional gains observed in this population. However, while both a single exercise bout (Miyauchi et al. 2003) and chronic exercise training (Johnson et al. 2001) increase pulmonary eNOS and NO signalling in healthy animals, their mechanism of action and impact in PAH have not yet been investigated. We therefore used a well-established model of monocrotaline (MCT)-induced PAH in rats to interrogate the immediate effects of a single bout of aerobic exercise on pulmonary artery (PA) pressures and pulmonary eNOS activity, as well as acute RV responses to exercise. The focus on RV responses is prompted by the need to address an important concern that exercise may accelerate detrimental RV remodelling in PAH, and therefore, the onset of right heart failure (Handoko et al. 2009). Such outcomes may largely depend upon RV wall stress during exercise bouts. Mild elevations in RV wall stress during exercise are well tolerated, occurring even in healthy subjects, due to increases in PA pressures and RV afterload. However, the magnitude of the RV wall stress response during exercise may be exaggerated in PAH, because both the resting PA pressures are higher and their rise relative to workload is steeper than in normal conditions (Janicki et al. 1985). In left ventricular (LV) failure, even a transient elevation in LV wall stress triggers detrimental pro-inflammatory responses in the myocardium (Sun et al. 2007). It is therefore important to establish RV wall stress responses to exercise in PAH and to determine optimal levels of exercise, i.e. tailored relative to individual aerobic capacity as a percentage of measured maximal rate of oxygen uptake (${\dot{V}}_{{\text{O}}_{\text{2}}\text{max}}$), that do not exacerbate RV wall stress responses beyond homeostatic levels.

A limitation of studies of pulmonary haemodynamic responses to exercise is reliance on single measurements performed on anaesthetized animals shortly after exercise cessation. Novel technological advancements in implantable telemetry may provide new insight into pulmonary haemodynamics during exercise and over a longer time span during disease development. Therefore, we include in this report additional observations enabled by the successful implantation of a telemetry probe and long-term live recording of pulmonary haemodynamics at rest and during exercise, in a PAH model. Our findings using this novel technique, as well as traditional technologies, indicated that exercise at moderately high relative intensity acutely lowers pulmonary vascular pressures in a model of PAH without causing detrimental effects in the RV and is consistent with a mechanism of heightened NO-dependent arterial relaxation.

## Methods

### Ethical approval

The experimental protocol was approved by the Institutional Animal Care and Use Committee of Indiana University, which is in compliance with National Iinstitutes of Health guidelines. All animals received care in compliance with the *Guide for the Care and Use of Laboratory Animals* 8th edition, Copyright 2011 by the National Academy of Sciences.

### Animals

Male Sprague–Dawley rats (~300 g; Charles River Chicago, Illinois, USA) were used in this study. All animals were housed in pairs in the Indiana University animal facility and fed standard rat chow and water *ad libitum*. The rats were maintained at an ambient temperature of 21–24°C with a 12 h–12 h dark–light cycle.

### Induction of PAH and experimental groups

Pulmonary arterial hypertension was induced once by administration of 50 mg kg^−1^ MCT (Sigma Aldrich) i.p., which reliably induces stable PAH after 2 weeks (22). Control (CON) animals received vehicle (saline) I.P. Animals were assigned to one of the following four treatment groups: (i) PAH plus acute exercise (PAH-Ex; *n* = 6); (ii) PAH but no exercise (PAH-UnEx; *n* = 6); (iii) control plus acute exercise (CON-Ex; *n* = 4); or (iv) control without exercise (CON-UnEx; *n* = 4).

### Treadmill familiarization

**Figure 1.**
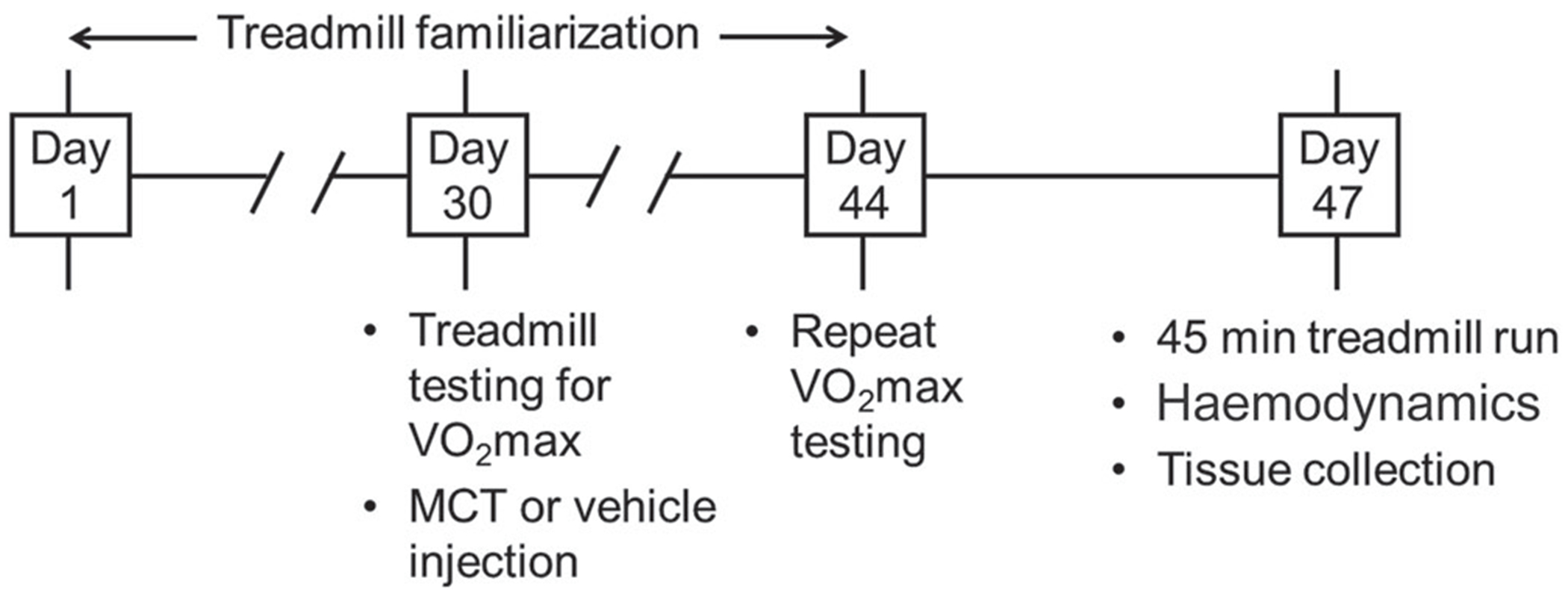
Study protocol After a period of treadmill familiarization, rats underwent exercise testing on a motorized treadmill for measurement of maximal oxygen uptake (${\dot{V}}_{{\text{O}}_{\text{2}}\text{max}}$). Rats then received either monocrotaline (MCT; 50 mg kg^−1^, i.p.) to induce pulmonary arterial hypertension (PAH; *n* = 12), or saline, for healthy control animals (*n* = 8). Two weeks after MCT/saline administration, ${\dot{V}}_{{\text{O}}_{\text{2}}\text{max}}$ testing was repeated. Three days after the second ${\dot{V}}_{{\text{O}}_{\text{2}}\text{max}}$ test, half of the rats from each group performed a single 45 min run, with treadmill workload set to elicit 75% of individually tested oxygen uptake reserve (${\dot{V}}_{O_{2}}\text{R}$), and the remaining rats rested on a stationary treadmill 45 min. Immediately after the 45 min run/rest period, haemodynamic measures were performed under general anaesthesia, followed by killing and tissue harvest.

As running on treadmill is a skilled activity for rats, all rats were familiarized with the treadmill (Columbus Instruments, Columbus, OH, USA) at speeds and inclines that would be required during subsequent treadmill testing. Familiarization was performed three times per week at speeds of 6–15 m min^−1^ and inclines of 0–15 deg. A mild electric stimulus at the back of the treadmill chamber promoted learning of running behaviour. The duration of one session each week was gradually increased over the familiarization period to up to 30 min to prepare for the final continuous run. The duration of the other two weekly familiarization sessions was kept to 5 min to minimize chronic training effects. In addition to serving as a customization period for rats to treadmill running, the familiarization protocol permitted evaluation of each rat’s innate willingness to run to facilitate later assignment of animals to acute exercise *versus* unexercised groups. Any animal that was unable to run consistently at the front of the treadmill by the end of the familiarization sessions was deemed unwilling. Only rats that were willing to run were randomized into the two exercise groups. Figure 1 presents a schematic diagram of the protocol time line.

### Maximal aerobic capacity testing

Immediately before the MCT/saline injections, exercise testing was performed on PAH-Ex and CON-Ex rats to determine maximal aerobic capacity (${\dot{V}}_{{\text{O}}_{\text{2}}\text{max}}$; expressed relative to body weight). Exercise testing was performed using an enclosed treadmill chamber that permits metabolic measurements via indirect open-circuit calorimetry (Oxymax; Columbus Instruments). Gas analyser calibrations were conducted before testing using standardized gas mixtures (Praxair, Indianapolis, IN, USA). Baseline measurements were collected until the rate of O_2_ consumption (${\dot{V}}_{O_{2}}$) stabilized (~5 min). The ${\dot{V}}_{O_{2}}$ was recorded every 30 s. For determination of ${\dot{V}}_{{\text{O}}_{\text{2}}\text{max}}$, a modified incremental treadmill protocol in 3 min stages was used (Bedford et al. 1979). The test was terminated when ${\dot{V}}_{O_{2}}$ plateaued despite increasing workload (Bedford et al. 1979) or when the rat was unable to maintain its position on the belt and received three consecutive electrical stimuli without recovery to the front of the treadmill. The highest ${\dot{V}}_{O_{2}}$ achieved was recorded as ${\dot{V}}_{{\text{O}}_{\text{2}}\text{max}}$ . In order to measure effects of PAH development on exercise capacity, ${\dot{V}}_{{\text{O}}_{\text{2}}\text{max}}$ determination was repeated 2 weeks after MCT injection. During the 2 weeks between injection and repeat ${\dot{V}}_{{\text{O}}_{\text{2}}\text{max}}$ testing, familiarization running was continued for three sessions per week (limited to 5 min per session). In order to evaluate the effects of acute exercise on haemodynamics and RV function, a single continuous treadmill run occurred 2 days later in PAH-Ex and CON-Ex animals. This was followed immediately by haemodynamic assessment and harvesting of tissues (see next section for specific experimental protocol). The 2 day time frame was chosen to allow for adequate recovery from ${\dot{V}}_{{\text{O}}_{\text{2}}\text{max}}$ testing.

### Single continuous treadmill run

After determination of body weight, rats were placed in the treadmill chamber. The PAH-Ex and CON-Ex groups performed a single 45 min run at a speed and incline that elicited 75% of ${\dot{V}}_{O_{2}}$ reserve (${\dot{V}}_{O_{2}}\text{R}$) determined in the second exercise test, calculated in the method of Karvonen as follows: $\left[\left({\dot{V}}_{{\text{O}}_{2}\max}-{\dot{V}}_{{\text{O}}_{2}}\text{resting}\right)\times 0.75\right]+{\dot{V}}_{{\text{O}}_{2}}$ resting. The intensity of 75% ${\dot{V}}_{O_{2}}\text{R}$ was chosen because it corresponds to the upper end of the exercise intensity range recommended by the American College of Sports Medicine for exercise prescription in cardiopulmonary patients (Thompson, 2010). Rats assigned to the PAH-UnEx and CON-UnEx groups were also placed in the treadmill chamber for 45 min, but the treadmill belt remained stationary. Immediately after the 45 min period, rats from the four experimental groups were prepared for invasive measures to follow.

### Invasive haemodynamic measurements

Immediately after their 45 min continuous run or stationary treadmill exposure, rats were anaesthetized by inhalation of isofluorane (5%), orotracheally intubated and mechanically ventilated under isofluorane maintenance (2.5%). The left carotid artery was cannulated with PE-50 tubing and the right internal jugular vein with a 2F Millar catheter (Millar Instruments, Houston, TX, USA) as described previously (Lahm et al. 2012). Surgery was performed on a servo-controlled heated tray that maintained animal temperature at 37°C. Recordings of pulmonary and systemic pressures were achieved between 35 and 45 min following transfer out of the treadmill chamber. Right ventricular systolic pressure (RVSP) and mean arterial pressure (MAP) were assessed in room air during normocapnia and normal pH. In order to account for effects of systemic blood pressure on RVSP, the RVSP was normalized for MAP and expressed as RVSP/MAP.

### Implantable telemetry

In order to assess haemodynamic changes during development of MCT-induced PAH and during treadmill running, we developed a method to measure exercise haemodynamics via implantable telemetry (Data Sciences International, Minneapolis, MN, USA), thus allowing for assessment of haemodynamics before and in the weeks after MCT injection within the same animal. A new-to-market small animal dual-pressure telemetry transmitter (model HD-S21; Data Sciences International) was used for these studies, which permitted simultaneous systemic and PA pressure measures, in addition to monitoring of ECG, heart rate (HR) and body temperature. The fully implanted transmitter (8 g) uses two fluid-filled sensing catheters (10 cm long, external diameter 0.7 mm) to signal to a remote receiver, calibrated by the manufacturer before use. Surgical implantation was performed in a healthy male Sprague–Dawley rat (280 g) using a sterile preparation, mechanical ventilation with isofluorane anaesthesia and a servo-controlled heated tray to maintain body temperature. The first catheter was inserted into the abdominal aorta via a mid-line abdominal incision, with the transmitter body placed in the abdomen. Leads for detection of ECG were tunnelled subcutaneously into the thorax and placed above the pectoral muscles. Placement of the second catheter for measurement of PA pressure used a thoracotomy approach similar to that previously described for a single pressure probe (Handoko et al. 2008). In brief, the catheter was fed from the abdomen through the intercostal space, inserted into the RV through a superficial purse-string suture (6–0 Prolene) and advanced into the PA. Telemetry readings during surgery indicated correct catheter placement before securing. The abdominal and chest cavities were closed using sutures and staples, and the animal recovered for 2 weeks before initial treadmill testing and subsequent MCT administration. The MCT dose for this animal was 40 mg kg^−1^ instead of 50 mg kg^−1^, to minimize risk of death before completion of the planned longitudinal study. Based on a recent report by Ruiter et al. (2013), it was suspected that PAH induced by MCT at this lower dosage may begin to resolve over time. Therefore, studies were conducted as late as 11 weeks after MCT injection, to document whether potential resolution occurred. Exercise testing for ${\dot{V}}_{{\text{O}}_{\text{2}}\text{max}}$ determination and the 45 min continuous treadmill run at 75% ${\dot{V}}_{O_{2}}\text{R}$ were thus performed at 2, 7 and 11 weeks after MCT injection. Recordings of systemic blood pressure (femoral aorta), PA pressures, HR, ECG and body temperature were obtained at rest and during all treadmill testing, as well as during recovery from testing. Oxygen pulse (in millilitres per minute), as a surrogate for stroke volume based on a rearrangement of the Fick equation (Whipp et al. 1996), was calculated as follows: ${\dot{V}}_{O_{2}}$ (in millilitres per minute)/HR (in beats per minute). Transmitter signals were relayed from the receiver to a data-exchange matrix connected to a computer equipped with Dataquest A.R.T. Gold 4.33 (Data Sciences International) software.

### Determination of right ventricular hypertrophy

Right ventricular hypertrophy was assessed by measuring the Fulton index [weight of the RV divided by weight of the left ventricle plus septum (S); RV/(LV + S)] as described previously (Lahm et al. 2012), with a value >~0.30 expected in the MCT-induced rat model of PAH. Immediately after determination of RV and LV + S weights, sections of the RV were snap-frozen for further biochemical analyses or immersed in 10% buffered formalin for immunohistochemical studies.

### Tissue harvest

Immediately after the haemodynamic measurements, rats were killed under general anaesthesia (inhaled isofluorane (5%)) via exsanguination. Harvested tissues from saline-perfused heart and right lung, saline- and agarose–formalin-perfused left lung and skeletal muscle samples (soleus) were preserved with cryofixation or formalin fixation and paraffin embedding as appropriate. Cryofixed tissues were stored at −80°C until biochemical assays were performed. For consistency, tissues from the telemetry-implanted rat were not included in the morphometric and biochemical assays.

### Pulmonary vascular remodelling

To characterize the pulmonary vascular phenotype, formalin-fixed, paraffin-embedded lung sections were heated, hydrated, treated for antigen retrieval, blocked with goat serum blocking buffer and incubated overnight at −4°C with rabbit polyclonal anti-smooth muscle actin (SMA, FisherSci PA1-37024; Fisher Scientific, Pittsburg, PA, USA) applied at 1:100 dilution. Following rinses, FITC goat anti-rabbit Ig (Invitrogen, Carlsbad, CA, USA) secondary antibody was applied at 1:200 dilution for 60 min at room temperature before rinsing, co-staining with 4′,6-diamidino-2-phenylindole (DAPI), and coverslip application (Vectashield; Vector Laboratories, Inc. Burlingame, CA, USA). The thickness of SMA-stained media was quantified using ImageJ software (NIH; Baltimore, MD, USA) in the following manner. Wall thickness measurements were obtained at four vessel locations on opposite sides of the vessel (WT1, WT2, WT3 and WT4), and vessel diameter (*D*) was measured as the longest diameter. Final wall thickness was expressed as a percentage of the vessel diameter using the following calculation: percentage wall thickness = {[(WT1 + WT2)100/*D*] + [(WT3 + WT4)100/*D*]}/2.

### Assessment of RV and skeletal muscle biochemical changes

In order to characterize the MCT-induced PAH phenotype further and to evaluate the acute RV inflammatory and pro-apoptotic responses to exercise, quantitative immunofluorescence microscopy was also performed on cryofixed soleus and/or RV tissue.

#### Density of glucose transporter-1 (Glut-1).

The density of Glut-1, an indicator of cytoplasmic glycolysis, was measured in cryofixed RV and soleus tissue (Archer et al. 2013) by incubating with anti-rabbit polyclonal Glut-1 (Abcam #ab652; Cambridge, MA, USA) at 1:150 dilution. Cryosections (7 *μ*m thick) were fixed in 10% paraformaldehyde, blocked with 3% bovine serum albumin blocking solution and incubated overnight at 22°C with primary antibodies. For detection of RV inflammation, cryosections were incubated with primary monoclonal antibodies for CD45 or CD68 (Santa Cruz Biotechnology, Dallas, TX, USA; 1:20 dilution). Following rinses, Alexa Fluor 568 anti-rabbit secondary antibody (Invitrogen) was applied at 1:200 dilution for 60 min at room temperature. After rising, sections were incubated with wheat germ agglutinin conjugated to Oregon Green-488 (for cell membrane staining; 5 *μ*g ml^−1^ in PBS, 10 min; Invitrogen) and DAPI (1 *μ*M in PBS; 10 min; Invitrogen), rinsed and coverslipped. Slides were imaged using immunofluorescence microscopy at ×20 magnification. Imaging thresholds were set to detect staining above autofluorescence levels, as determined by negative controls. The mean pixel intensity of Glut-1 staining and the relative number of CD45^+^ and CD68^+^ cells were determined using ImageJ software (NIH). The CD45^+^ and CD68^+^ counts were expressed as the number of positive stained cells per field, averaging at least six randomly chosen fields per RV.

#### Right ventricular cardiomyocyte apoptosis.

Right ventricular cardiomyocyte apoptosis was assessed with two distinct methods, namely terminal deoxynucleotidyl transferase dUTP nick end labelling (TUNEL) of RV cryosections and an activity assay for caspase-3 (Promega, Madison, WI, USA) in RV homogenates. TUNEL was performed on cryosections according to the manufacturer’s instructions (Roche Applied Science, Indianapolis, IN, USA) with DAPI co-staining, and the number of TUNEL-positive nuclei was expressed as normalized by total number of nuclei. The activity assay for caspase-3 in RV homogenates was performed as previously described (Petrache et al. 2006).

### Assessment of lung eNOS activation

Measurement of lung total eNOS and eNOS phosphorylated at serine 1177 or at threonine 495 sites (p-eNOS^Ser1177^ and p-eNOS^Thr495^, respectively) was performed via electrophoresis and immunoblot analysis of lung homogenates. The protein concentration was determined by standard bicinchoninic acid protein assay (BCA; Pierce, Rockford, IL, USA), and equal amounts of protein were resolved by 7.5% SDS-PAGE, followed by immunoblotting, as previously described (Lockett et al. 2014). Rat brain extract (Santa Cruz Biotechnology) was loaded as a positive control. Membrane probing was performed using a polyclonal antibody for eNOS (Santa Cruz Biotechnology; 1:200 dilution), p-eNOS^Ser1177^ (Cell Signaling, Danvers, MA, USA; 1:500 dilution), p-eNOS^Thr495^ (Cell Signaling; 1:500 dilution) or vinculin (Sigma Aldrich; 1:1000 dilution) as loading control. Following washes and incubation with horseradish peroxidase-conjugated anti-mouse or anti-rabbit IgG antibody (GE Healthcare, Buckinghamshire, England; 1:5000 dilution), eNOS, p-eNOS^Ser1177^, p-eNOS^Thr495^ and vinculin proteins were detected by the ECL system (Amersham Life Science, Pittsburgh, Pennsylvania). The intensity of Western blotting bands was measured by densitometry using ImageJ software (NIH) and expressed normalized to the vinculin band intensity.

### Statistical analyses

Data are presented as means ± SEM. Groups were compared by one-way ANOVA, using Tukey’s multiple comparison *post hoc* test analysis to determine between-group differences. Differences at an *α*-level of 0.05 (*P* < 0.05) were considered statistically significant.

## Results

### Impact of MCT-induced PAH on exercise capacity and on the metabolic activity of RV and skeletal muscles

**Figure 2.**
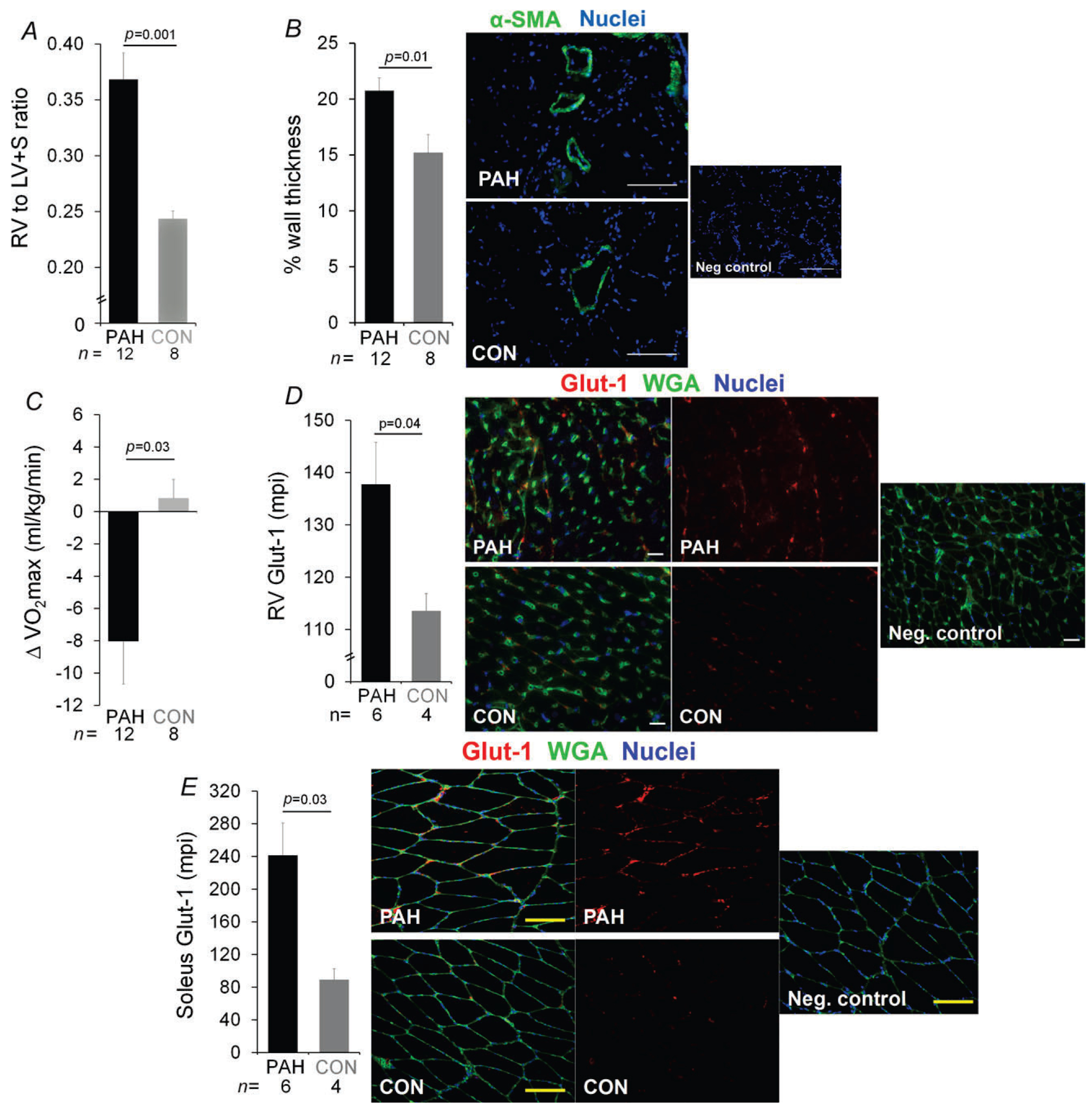
Phenotype of PAH (black bars) after MCT (50 mg kg^−1^) *versus* vehicle (CON; grey bars) *A*, Fulton index, indicator of right ventricular (RV) hypertrophy [RV mass/left ventricular (LV) + septal (S) mass; shown as means + SEM]. *B*, wall thickness (percentage of pulmonary arterial area; means ± SEM) measured by smooth muscle actin (*α*-SMA) immunofluorescence staining of lung shown in adjacent panels in green, with blue representing nuclei. *C*, change in aerobic capacity from baseline (in millilitres per kilogram per minute; shown as means + SEM) measured by ${\dot{V}}_{{\text{O}}_{\text{2}}\text{max}}$. *D* and *E*, glucose transporter-1 (Glut-1) expression [expressed as mean pixel intensity (mpi); shown as means + SEM; in RV and soleus harvested immediately after running, measured by immunofluorescence staining shown in red in adjacent panels, with green representing wheat germ agglutin-stained myocyte membrane and blue representing nuclei. Representative images are shown, with results from analysis of all animals depicted in bar graphs to the left of images. The number of animals for each group is shown under the bars. *P* Values are by one-way ANOVA.

As expected, MCT administration at a dose of 50 mg kg^−1^ resulted in a moderate PAH phenotype by 2 weeks postinjection, as evidenced by increases in Fulton index and PA muscularization (Fig. 2). Right ventricular weight relative to LV + S weight (Fulton index; Fig. 2A) indicated moderate RV hypertrophy in PAH compared with CON animals (0.37 ± 0.02 *versus* 0.24 ± 0.01, *P* = 0.001). Smooth muscle actin immunofluorescence staining of lung (Fig. 2B) indicated greater muscularization of pulmonary arteries in PAH compared with CON animals (percentage wall thickness = 20.8 ± 1.1 *versus* 15.2 ± 1.6, *P* = 0.01). The PAH rats exhibited a 14% decrement in aerobic capacity (Fig. 2C) from pre-injection ($\left(\Delta {\dot{V}}_{{\text{O}}_{2}\max}=-8.0\pm 2.6\text{ml}{\text{kg}}^{-1}\min^{-1}\right)$, whereas CON animals maintained their exercise capacity (+0.8 ± 1.2 ml kg^−1^ min^−1^, or +1.6 ± 2.1%, PAH *versus* CON; *P* = 0.03), suggesting a significant impairment in physical function in PAH rats. The PAH rats exhibited greater expression of glucose transporter Glut-1 in RV (Fig. 2D) and soleus (Fig. 2E), suggesting a shift toward non-oxidative glycolytic metabolism in both cardiac and skeletal muscle in diseased animals, which may explain their decreased exercise capacity.

### Effect of acute exercise on cardiopulmonary haemodynamics in PAH

**Figure 3.**
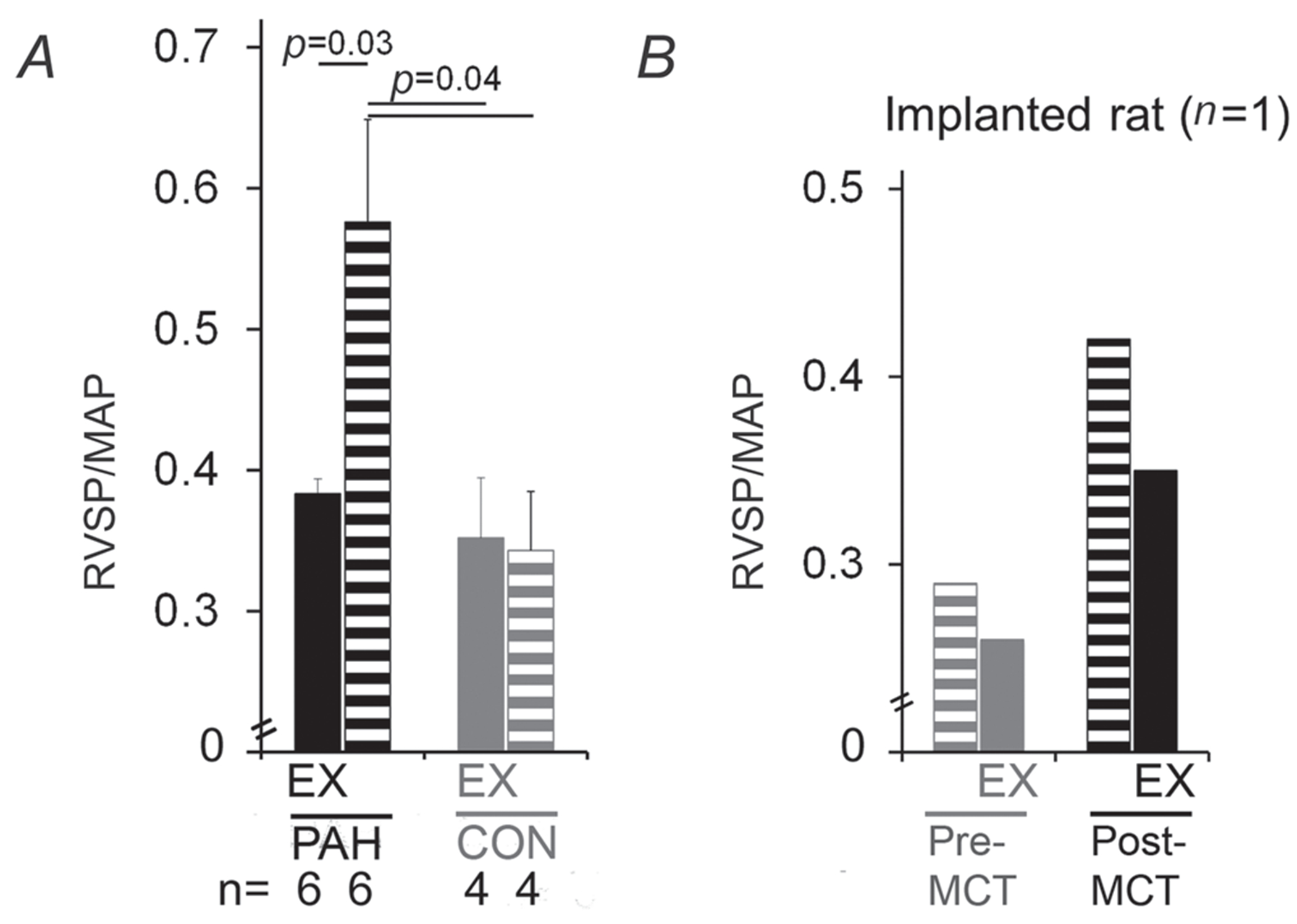
Haemodynamic responses to an acute bout of exercise *A*, right ventricular systolic pressure [RVSP, normalized by mean arterial pressure (MAP); shown as means + SEM] measured with a Millar catheter 30–40 min postexercise (EX, treadmill running 45 min at 75% ${\dot{V}}_{O_{2}}\text{R}$) for MCT (50 mg kg^−1^; PAH, filled black bars) and healthy rats (CON, filled grey bars) *versus* unexercised counterparts (striped black and grey bars); the number of animals for each group is shown under the bars. *P* Values are by one-way ANOVA. *B*, RVSP measured via implantable telemetry immediately before exercise (striped bars) and during recovery from exercise (EX, treadmill running 45 min at 75% ${\dot{V}}_{O_{2}}\text{R}$, filled bars) in an individual animal before (grey bars) and at 2 weeks post-MCT (40 mg kg^−1^) administration (black bars).

A single 45 min bout of running at 75% ${\dot{V}}_{O_{2}}\text{R}$ significantly lowered the RVSP (normalized by systemic MAP) in rats with PAH. This acute reduction in RVSP after exercise was not observed for healthy control rats (Fig. 3A).

### Effect of acute exercise on lung eNOS in PAH

**Figure 4.**
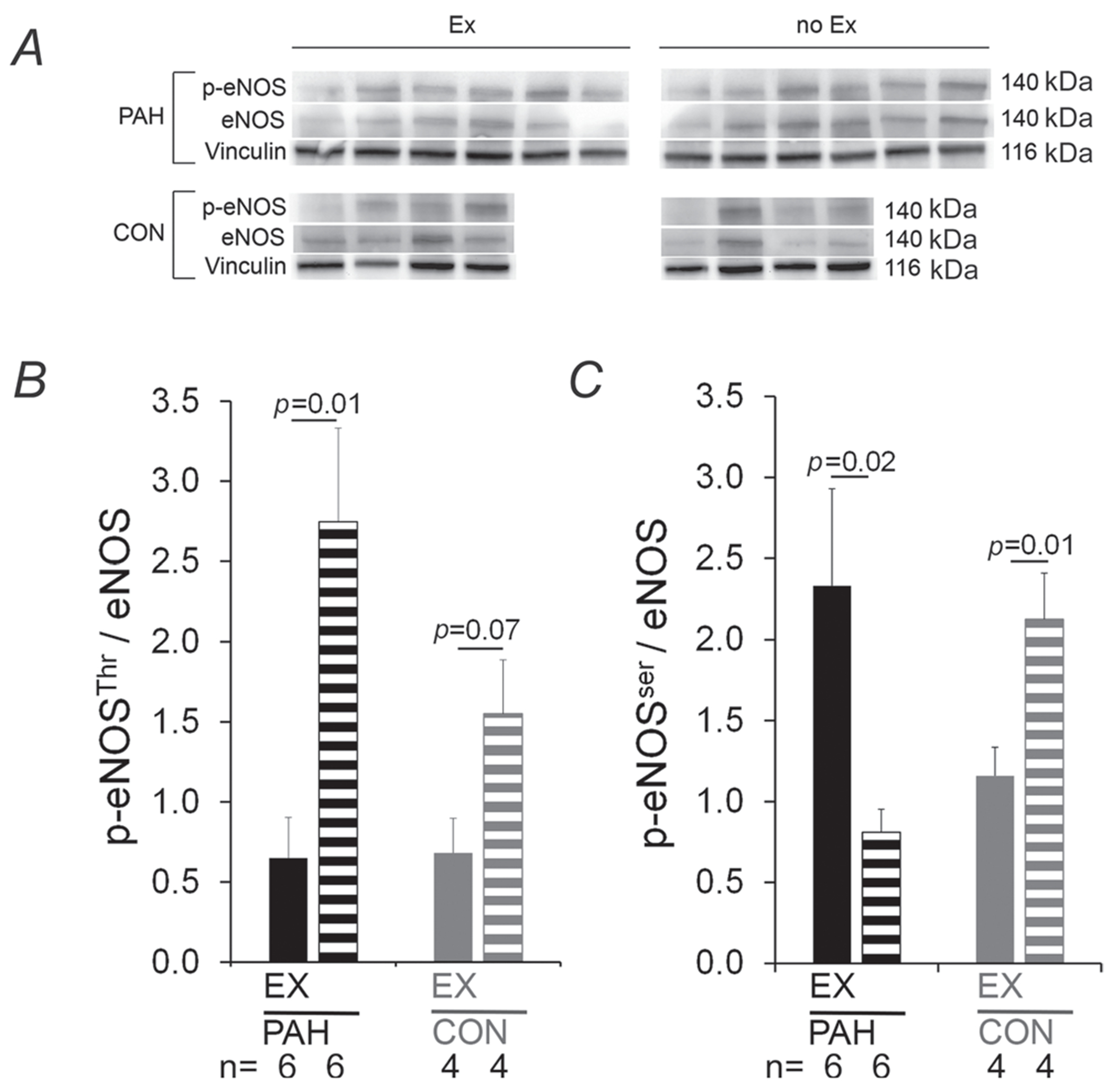
Endothelial nitric oxide synthase (eNOS) activation in acutely exercised PAH rats *A*, the immunoblot for lung homogenates with antibody against phosphor-eNOS (at Thr^495^, which inhibits eNOS activity), eNOS and vinculin, with lanes representing PAH (top panels) and healthy CON rats (lower panels) following acute exercise (45 min continuous treadmill run at 75% ${\dot{V}}_{O_{2}}\text{R}$, Ex) or no exercise (no Ex). Bar graphs indicate group means (+ SEM) measured by densitometry of immunoblots for phosphorylation of eNOS at Thr^495^ (*B*), which inhibits eNOS activity, and phosphorylation of eNOS at Ser^1122^ (*C*), which increases eNOS activity, for PAH EX (filled black bars) and healthy CON EX (filled grey bars) *versus* unexercised counterparts (striped bars). The number of animals for each group is shown under the bars. P-values are by one-way ANOVA.

To investigate whether acute exercise regulates the NO, a major regulator of pulmonary vascular tone, eNOS activation status was assessed by immunoblotting with phospho-eNOS specific antibodies of lung tissues from all experimental groups. In animals with PAH, acute exercise inhibited Thr495 phosphorylation (which inhibits eNOS activity) by 80% (*P* = 0.01; Fig. 4B), and activated Ser1177 phosphorylation (which activates eNOS) by 200% (*P* = 0.02; Fig. 4C). In healthy animals, acute exercise tended to decrease Thr495 phosphorylation (by 57%, *P* = 0.07) as expected, but unexpectedly decreased Ser1133 phosphorylation (by 45%, *P* = 0.01), inconsistent with published reports (Miyauchi et al. 2003), suggesting a different response of eNOS activity to acute exercise in healthy animals. In addition to effects on phosphorylation (activity), acute exercise increased total levels of lung eNOS protein in both PAH (0.83 ± 0.1 *versus* 0.5 ± 0.04 at rest; *P*=0.03; data not shown) and healthy animals (0.62 ± 0.08 *versus* 0.31 ± 0.09 at rest; *P* = 0.03; data not shown). Taken together, these data indicate that acute exercise increases pulmonary eNOS levels and activation in rats with MCT-induced PAH.

### Evaluation of impact of acute exercise in MCT-induced PAH on RV inflammation or apoptosis

**Figure 5.**
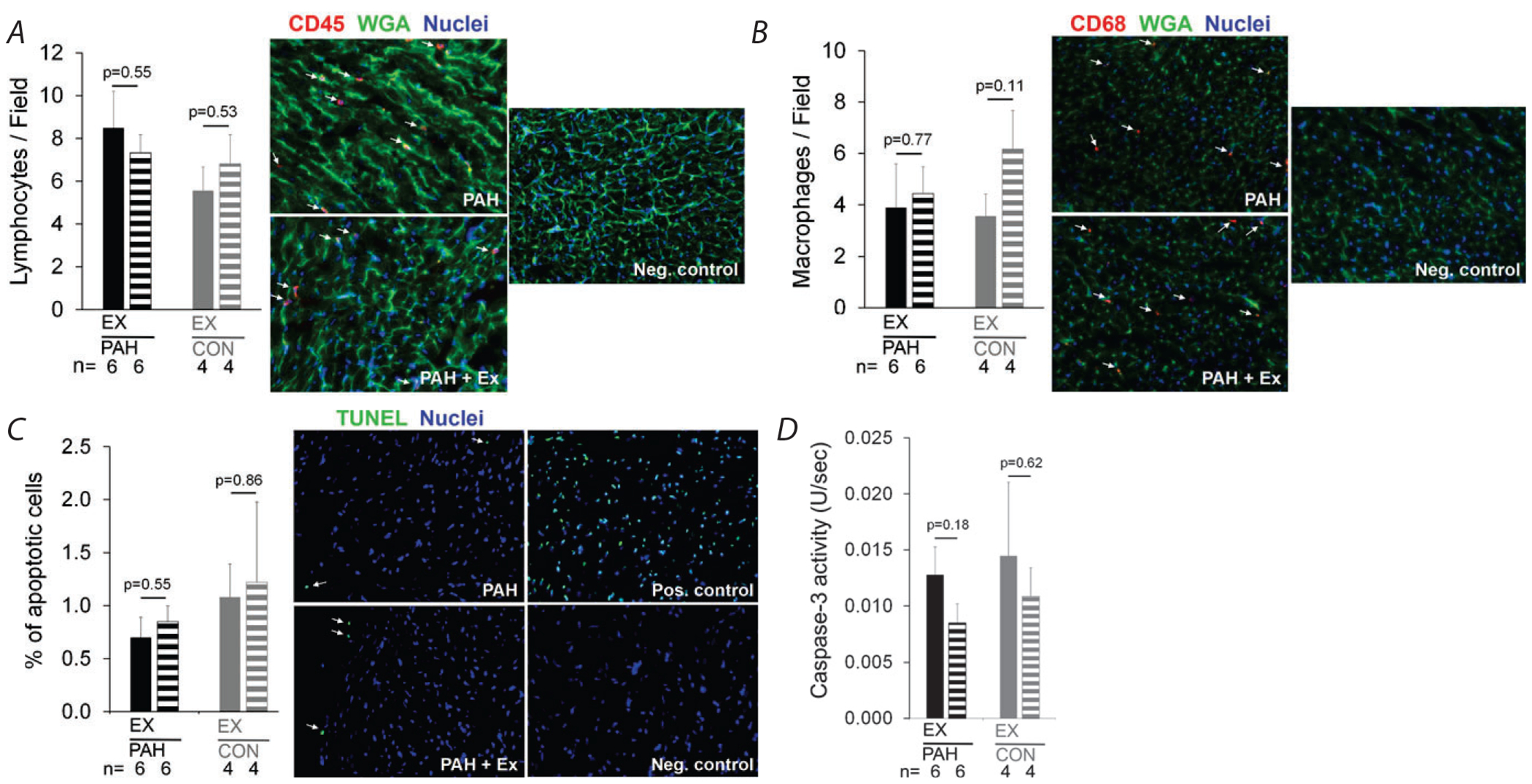
Testing for RV inflammation and apoptosis in acutely exercised PAH rats Infiltration of CD45^+^ cells (lymphocytes; *A*) and CD68^+^ cells (macrophages; *B*) measured by immunofluorescence staining (count per field; shown as means + SEM) of RV obtained after acute exercise (45 min continuous treadmill run at 75% ${\dot{V}}_{O_{2}}\text{R}$, EX, filled bars) or no exercise (striped bars) for PAH (black) and healthy CON rats (grey). Adjacent panels are representative images, with arrows indicating examples of CD45^+^ and CD68^+^ cells (red), green representing wheat germ agglutin-stained myocyte membrane, and blue representing nuclei. Right ventricular samples were also assessed for myocyte apoptosis by TUNEL staining (percentage of TUNEL^+^ cells; shown as means + SEM; *C*) and caspase-3 activity (shown as means + SEM; *D*). The number of animals for each group is shown under the bars. *P* Values are by one-way ANOVA.

To assess whether running at moderately high relative intensity may induce wall stress and therefore RV inflammation and apoptosis, we examined RV tissue harvested and cryofixed within 45 min of completion of the 45 min run at 75% ${\dot{V}}_{O_{2}}\text{R}$. Acute exercise did not increase RV inflammatory cells, including macrophage/monocyte abundance, as indicated by similar levels of CD45^+^ (Fig. 5A) and CD68^+^ (immunostaining Fig. 5B) in all groups tested. Furthermore, acute exercise did not trigger RV apoptosis, as indicated by similar TUNEL staining (Fig. 5C) and similar caspase-3 activity (Fig. 5D) in the RV tissues of all groups tested.

**Figure 6.**
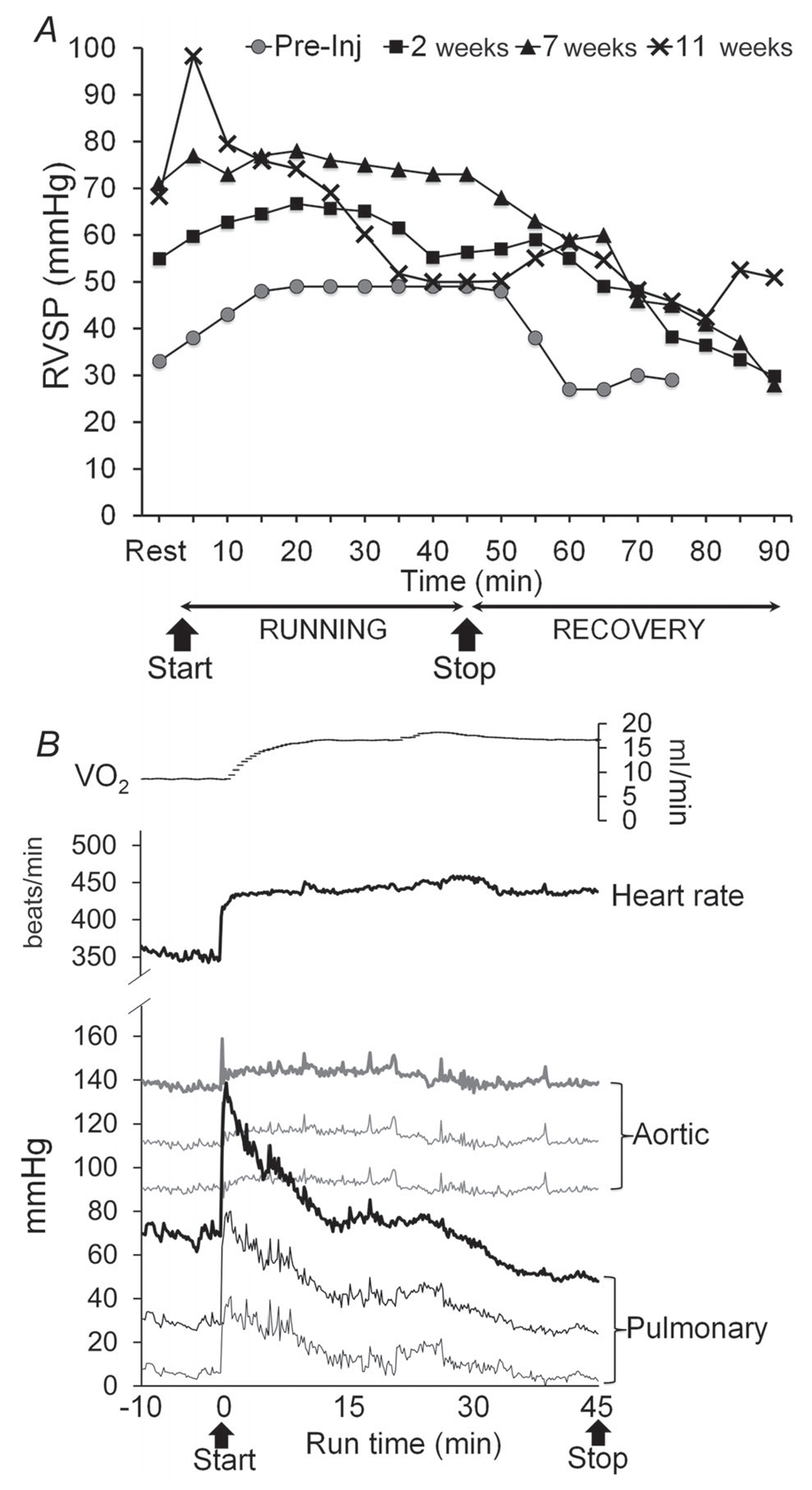
Exercise haemodynamics assessed longitudinally in a PAH model via serial, real-time telemetric recordings *A*, RVSP kinetics during exercise and postexercise (continuous treadmill running at 75% ${\dot{V}}_{O_{2}}\text{R}$) in an individual animal measured via implantable telemetry before (grey circles) and after MCT injection (40 mg kg^−1^; black symbols) at 2 (squares), 7 (triangles) and 11 weeks (crosses). *B*, oxygen uptake (${\dot{V}}_{O_{2}}$), heart rate and blood pressure traces (systolic, mean and diastolic) for the systemic (aortic) and pulmonary circulations recorded via implantable telemetry during continuous treadmill running (75% ${\dot{V}}_{O_{2}}\text{R}$) 11 weeks after MCT (40 mg kg^−1^) administration.

In summary, data obtained by traditional cross-sectional analysis of a large group of animals indicated a significant beneficial effect of acute exercise on outcomes of cardiopulmonary performance in PAH, without detrimental side-effects. However, longitudinal data on the effects of acute exercise within an animal could not previously be assessed. Therefore, using implantable telemetry, we had the opportunity to perform serial recordings of previously unreported pulmonary haemodynamic responses during disease onset and during exercise within the same animal. We used this technique as a proof-of-principle validation of cohort cross-sectional data. As expected, this technique validated the model of PAH, because after administration of MCT (40 mg kg^−1^), resting PA pressures increased significantly compared with baseline levels (Figs 3B and 6A). It also made feasible the recording of PA pressures during acute exercise bouts in PAH, showing that they were augmented during exercise (Figs 3B and 6A). However, at least in this proof-of-principle experiment, PA pressures in PAH quickly returned to baseline during early recovery from exercise, the time period ‘missed’ due to surgical procedures required for invasive measures, and after 20 min, decreased even further to below baseline values (Figs 3B and 6A). Eventually, these PA pressures recovered to a stable baseline 1.5–2 h postexercise (data not shown), which is in contrast to pre-MCT (physiological) PA pressure responses that return to baseline at ~20 min postexercise (Fig. 6A). Interestingly, the time to onset of this exaggerated decline in PA pressure postexercise decreased the longer the measurement was after MCT administration (Figs 3B and 6A). Moreover, at 11 weeks post-MCT (Fig. 6A), not only were postexercise PA pressures lower than resting values, but even during the exercise the PA pressures declined to levels lower than resting after ~25 min of continuous running at unchanged intensity (75% ${\dot{V}}_{O_{2}}\text{R}$). This was accompanied by a stable oxygen pulse (range 0.039–0.040) mL/min, indicating that the reduction in PA pressures observed does not represent RV pump failure, corroborating the data in the rat cohort showing improved RVSP after acute exercise. Furthermore, as implantable telemetry in the instrumented rat was capable of uninterrupted recordings of haemodynamics during the exercise-to-recovery transition, it was possible to capture the time period ‘missed’ due to surgery for invasive measures (35–45 min postexercise) in the rat cohort. The real-time traces of resting, exercise and recovery pulmonary pressures are shown in Fig. 6B, along with simultaneous systemic pressures and HR and ${\dot{V}}_{O_{2}}$ measures. In aggregate, these data indicate the feasibility of real-time telemetry in rats with PAH, corroborate the longitudinal PA pressure changes with the changes in eNOS signalling in the rat cohort and support a beneficial cardiopulmonary effect of acute exercise in rats with MCT-induced PAH.

## Discussion

The most important finding of this study is that a single bout of exercise can transiently normalize PA pressure in MCT-induced PAH and is associated with increased lung eNOS activation, supporting a mechanism of acute NO-mediated pulmonary vasodilatation. In healthy individuals, exercise is known to promote an acute reduction in pulmonary vascular resistance, secondary to vasodilatation of resistance vessels as well as pulmonary vascular recruitment (Kovacs et al. 2009; Bossone & Naeije, 2012; Lewis et al. 2013; Naeije et al. 2013). However, no studies to date have examined the acute exercise response in PAH animal models. Previous studies of chronic exercise (exercise training) effects in animals and in patients with PAH revealed mixed findings regarding the impact on haemodynamics, with one study demonstrating a training-induced lowering of resting pulmonary pressures (Grünig et al. 2012), but other studies indicating no effect (Mereles et al. 2006; Fox et al. 2011).

Our haemodynamic measures suggest that for exercise at the same relative intensity, the acute pulmonary vasodilatory response may be enhanced in MCT-induced PAH. This implies a dominant vasoconstrictive component to the elevated pulmonary pressures at rest and during exercise in this model, which may be relieved acutely by exercise-induced sheer stress-mediated pulmonary eNOS activation and heightened NO production. Given that flow-induced sheer stress at the pulmonary endothelium is proportional to resistance, the reduction in PA pressures for MCT rats measured postexercise may simply be a consequence of a greater upsurge in pulmonary pressures at the onset of exercise, which would invoke even greater flow-mediated sheer stresses to the pulmonary endothelium. As endothelial sheer stresses result in a burst of NO production in a manner that is more dependent on temporal gradients than the absolute magnitude of sheer stress (Kuchan & Frangos, 1994), it is fitting that more pronounced exercise-induced PA pressure reduction along with greater eNOS activation occurred for MCT compared with healthy rats exercised at the same relative intensity and duration.

In order to optimize an exercise prescription for PAH, we require a better understanding not only of exercise haemodynamics but also of potentially unintended adverse effects of exercise, including those induced acutely by right wall stress during exercise bouts. Therefore, we examined the RV myocardium for evidence of pro-inflammatory/apoptotic signalling after the bout of treadmill running. While the acute myocardial responses to exercise in PAH have not been investigated previously, Handoko et al. (2009) reported widespread leucocyte infiltration of the RV as well as accelerated pulmonary vascular remodelling in response to chronic training for rats with a more severe and progressive form MCT-induced PAH (induced by 60 mg kg^−1^), suggesting that RV wall stress may have been detrimentally elevated during exercise bouts for these rats. In contrast, our histological and biochemical assays did not indicate RV tissue inflammatory cell infiltration or apoptosis following an acute bout of exercise in either healthy or PAH rats. In addition to differences in the severity of the PAH disease model and the duration of exercise, the disparate results may be explained by the absolute intensity of exercise. In the study by Handoko et al. (2009), all animals were exercised at the same absolute workload, which could have led to a greater relative strain on the animals with PAH. In contrast, our rats performed exercise at a prescribed relative exercise intensity (a percentage of tested maximum) rather than absolute workload. This not only facilitates between-group comparisons but also improves translation of dose–response observations in the rat model, ultimately, to an optimized exercise training prescription for patients with PAH, whose exercise intensities are always prescribed relative to individual tolerances. Future studies will determine whether the relative exercise intensity applications will have a similar beneficial effect on PA pressures with no detrimental RV effects in PAH induced by higher MCT concentrations or in other models of PAH.

Additional insight obtained from measurements made during exercise using novel implantable telemetry over the time course of PAH development within the same animal corroborate findings from the cross-sectional analysis of the larger group, and additionally, suggest that exercise haemodynamics may be disease-phase dependent. For example, while absolute values were higher following PAH induction, the change in RVSP with exercise differed between 2, 7 and 11 weeks post-MCT and raises the question of whether disease stage-dependent variances in pulmonary vasoresponsiveness and/or RV dysfunction during exercise underlie this observation, as has been suggested for patients (Tolle et al. 2008). Given that the pattern of haemodynamic response to exercise may relate better to RV function than resting haemodynamics in PAH patients (Tolle et al. 2008) and also serve potential prognostic value, this underscores the need for continued evaluation of exercise haemodynamics in PAH animal models, such as we have introduced here. An additional point of interest raised from the haemodynamics recorded serially in the instrumented animal is that the time point with the most marked exercise-induced pulmonary vasorelaxation (11 weeks after 40 mg kg^−1^ MCT) corresponded to the time frame recently suggested to represent potential reversibility of a mild (40 mg kg^−1^ dosage) MCT-induced PAH (Ruiter et al. 2013). Further investigation is warranted to determine whether this so-called ‘phenotype reversal’ in mild MCT-induced PAH might represent amelioration of the pulmonary vasoconstriction component of the model.

### Limitations and future directions

The changes in eNOS activation with acute exercise in PAH suggest that this may be a preferred mechanism for vasorelaxation, a hypothesis that can be tested with future loss-of-function studies and when investigating approaches to optimize chronic exercise adaptations for PAH. Such mechanistic studies are likely to be enhanced by complementing end-point cross-sectional traditional analyses with novel dual-pressure implantable telemetry recordings during exercise, as we have introduced here. Given the significant material and skilled technical resources required with any novel technology, we have provided only proof-of-principle data using a single animal that dual-pressure implantable telemetry is useful and relatively safe in a PAH model, and corroborates data obtained via rigorously powered cohort studies. As illustrated by our traces, such technique will probably be useful for the investigation of disease-severity dependence of exercise haemodynamics in this and other models of pulmonary vascular disease and for monitoring the response to therapeutic interventions in PAH. Finally, another limitation of our study is that, while stroke volume estimations during exercise and postexercise biochemical assays indicated a neutral RV response to exercise, methodology was not available to assess RV function directly in response to exercise. Further investigations using postexercise echocardiography, or future implantable telemetry technology that permits direct cardiac output measurements, will provide a more rigorous assessment of the RV response to exercise in PAH.

Our report identifies, for the first time, that in rats with MCT-induced PAH, a single bout of continuous exercise at moderately high relative intensity induces a reduction in pulmonary artery pressure associated with lung eNOS activation, without promoting acute RV inflammation or cardiomyocyte apoptosis. These favourable adaptations suggest that appropriate, individualized exercise regimens should be investigated as potential adjunct therapeutic interventions in PAH. Examination of pulmonary pressure responses to various exercise approaches may reveal an optimal protocol to maximize sheer stress-mediated pulmonary eNOS activation, as well as a during- or postexercise ‘window’ of PAH alleviation, as suggested by our findings. Implementation of such a protocol in chronic training would provide sufficient stimulus for positive exercise adaptations while reducing the total right heart stress from cumulative exercise bouts.

## New Findings

- What is the central question of this study?

The acute effect of exercise at moderately high intensity on already-elevated pulmonary arterial pressures and right ventricular wall stress in a rat model of pulmonary arterial hypertension (PAH) is unknown.

- What is the main finding and its importance?

We show, for the first time, that in a rat model of PAH, exercise induces an acute reduction in pulmonary artery pressure associated with lung endothelial nitric oxide synthase activation, without evidence of acute right ventricular inflammation or myocyte apoptosis. Haemodynamic measures obtained with traditional invasive methodology as well as novel implantable telemetry reveal an exercise-induced ‘window’ of pulmonary hypertension alleviation, supporting future investigations of individualized exercise as therapy in PAH.
